# Molecular and mechanistic insights into the gut-liver axis in *K. pneumoniae* infections: current advances and future directions

**DOI:** 10.1128/iai.00477-25

**Published:** 2026-03-04

**Authors:** Jie Deng, Ling Cheng, Jinbo Liu, Zhangrui Zeng

**Affiliations:** 1 Department of Laboratory Medicine, The Affiliated Hospital, Southwest Medical University 556508, Luzhou, China; 2 Sichuan Province Engineering Technology Research Center of Molecular Diagnosis of Clinical Diseases, Luzhou, China; 3 Molecular Diagnosis of Clinical Diseases Key Laboratory of Luzhou, Luzhou, China; 4 Department of Healthcare-associated Infection Control, The Affiliated Hospital of Southwest Medical University 556508, Luzhou, China; University of Pittsburgh, Pittsburgh, Pennsylvania, USA

**Keywords:** bacterial translocation, gut-liver axis, immune mechanism, inflammatory response, intestinal barrier

## Abstract

*Klebsiella pneumoniae* is an opportunistic Gram-negative pathogen in clinical settings, primarily causing opportunistic infections in hospitals. The prevalence of liver abscess cases caused by hypervirulent *K. pneumoniae* (hvKP) has significantly increased in recent years, renewing interest in the interaction between this bacterium and the gut-liver axis. Research has shown that *K. pneumoniae* disrupts intestinal barrier integrity by adhering to and producing virulence factors, leading to bacterial translocation to vital organs such as the liver. This activation of inflammatory signaling pathways triggers systemic infection, exacerbating liver damage and systemic inflammation. The current research still faces key challenges: the molecular mechanism of bacteria crossing the intestinal barrier to reach the liver is not yet clear, the dynamic regulatory network of the gut-liver axis during infection lacks systematic analysis, and insufficient clinical research data limit a comprehensive understanding of its pathogenic mechanism. This review systematically organizes the latest research progress on the interaction between *K. pneumoniae* infection and the gut-liver axis, with a focus on the breakdown of the intestinal barrier mediated by virulence factors, mechanisms of bacterial translocation, and the collaborative regulatory network of immune cells (such as macrophages and neutrophils) and inflammatory pathways in the gut-liver axis. By analyzing the immune evasion strategies of hvKP and the host-pathogen arms race with host immune responses, it provides theoretical references for a deeper understanding of its pathogenic mechanism and lays the foundation for clinical development of precise intervention strategies targeting inflammatory pathways or intestinal barrier repair.

## INTRODUCTION

*K. pneumoniae*, a common Gram-negative rod bacterium, is widely present in the natural environment and the human gastrointestinal tract. It is both a normal part of the gut flora and an important opportunistic pathogen (1). In recent years, with the rapid spread of antibiotic-resistant strains (especially those producing extended-spectrum β-lactamases [ESBLs] and carbapenemase KPC), this bacterium has become a major threat in the global public health field (2). In the past, *K. pneumoniae* has primarily been associated with hospital-acquired pneumonia, but there has been a notable rise in the prevalence of community-acquired hypervirulent *K. pneumoniae* (hvKP) infections. This increase is concerning due to the pathogen’s high virulence and aggressiveness, which can result in serious conditions like liver abscesses and septicemia (3, 4).

HvKP is characterized by its enhanced ability to break through the intestinal barrier and its potential for extraintestinal dissemination, particularly targeting the liver and causing pyogenic liver abscess, a process closely related to the gut-liver axis (5). The gut-liver axis serves as a crucial bidirectional communication pathway connecting the gastrointestinal tract with the liver, regulating metabolic balance and immune homeostasis through the portal vein circulation, bile secretion, and neuroimmune network (6). In a healthy state, the gut microbiota maintains intestinal barrier integrity (such as tight junction proteins and the mucosal layer) and regulates immune cell function (such as macrophages and dendritic cells), forming a coordinated defense system with the liver to resist pathogen invasion (7). However, hvKP infection can disrupt this homeostasis. On one hand, its virulence factors promote colonization and dissemination by disrupting the intestinal epithelial barrier and evading intestinal immune rejection (8). On the other hand, bacteria that migrate to the liver activate pattern recognition receptor pathways, such as Toll-like receptors (TLRs) and NOD-like receptors (NLRs), triggering excessive inflammation and immune imbalance, exacerbating liver damage and systemic infection (9, 10).

Despite the preliminary findings in existing research revealing the destruction of the intestinal barrier by *K. pneumoniae* and its immune regulation mechanisms, there are still key scientific questions that urgently need to be clarified: How does hypervirulent *K. pneumoniae* precisely regulate the intestinal-liver immune network through virulence factors to achieve evasion and invasion? What are the dynamic immune regulatory mechanisms of the gut-liver axis during infection? How does the interplay between host immune responses and bacterial virulence affect the outcome of infection?

This review systematically summarizes recent research progress, focusing on the immune evasion strategies of *K. pneumoniae* (especially hvKP), the molecular mechanisms of intestinal barrier breach, and the collaborative regulatory network of immune cells and inflammatory pathways in the gut-liver axis. The aim is to provide theoretical references for a deeper understanding of the pathogenic mechanisms of this bacterium and to lay the foundation for the development of intervention strategies targeting immune pathways.

## BIOLOGICAL CHARACTERIZATION OF *K. PNEUMONIAE*

### Adhesion colonization of the bacterial gastrointestinal tract

*K. pneumoniae* is capable of colonizing the gastrointestinal tract, and its ability to persist in this niche represents a critical prerequisite for bacterial translocation and subsequent extraintestinal infections. Successful intestinal colonization relies on the coordinated action of bacterial surface structures, metabolic adaptability, and competitive advantages, enabling adhesion to host tissues, tolerance to environmental stresses, and overcoming colonization resistance imposed by the commensal microbiota (11).

Fimbriae constitute the core structures mediating adhesion, and *K. pneumoniae* expresses both type I and type III fimbriae, which play complementary roles during intestinal colonization. Type I fimbriae are encoded by the fim gene cluster and mediate mannose-sensitive adhesion by recognizing mannose-containing glycoproteins on host epithelial cells, representing a key factor in early urinary tract infection. In contrast, type III fimbriae are encoded by the mrk gene cluster and mediate mannose-resistant adhesion, primarily contributing to biofilm formation and enhancing persistent attachment to epithelial and abiotic surfaces, thereby improving bacterial survival in the intestinal environment (12–14). The coordinated expression of these two fimbrial types facilitates stable colonization and reduces the likelihood of bacterial clearance. Although the capsule polysaccharide (CPS) is not a primary adhesin, its thickness and surface properties can indirectly influence fimbrial exposure and bacteria-host interface interactions, thereby modulating colonization stability.

*K. pneumoniae* exhibits high metabolic plasticity, which enhances its competitive fitness within the intestinal ecosystem. The bacterium can exploit nutrients derived from host or commensal microbial metabolism, such as ethanolamine and fucose released from mucins, thereby circumventing direct nutritional competition with resident microbiota (15). This metabolic advantage becomes particularly pronounced following microbiota disruption or antibiotic treatment, facilitating sustained bacterial expansion. In addition, manganese homeostasis mediated by the manganese transporter (MntP) enhances resistance to oxidative stress, allowing *K. pneumoniae* to survive within inflammation-associated intestinal microenvironments (16).

In terms of competitive adaptation, genomic islands unique to hypervirulent *K. pneumoniae* encode Colibactin and chromosomally encoded type VI secretion system, which can directly suppress or eliminate competing intestinal microbes, thereby reshaping the local microbiota and promoting long-term colonization (17, 18). Dietary factors also influence intestinal colonization efficiency, as dietary fiber has been shown to restrict *K. pneumoniae* expansion, whereas specific carbohydrates such as lactulose promote intestinal overgrowth and systemic dissemination (19).

Collectively, *K. pneumoniae* establishes a stable ecological niche in the gastrointestinal tract through adhesion, nutritional competition, and metabolic adaptation, laying the foundation for intestinal barrier disruption and subsequent invasive infections. Stronger colonization capacity and prolonged persistence within the host are associated with an increased risk of infection and greater challenges in antimicrobial treatment.

### Pathogenesis and immune escape

The pathogenicity of *K. pneumoniae* involves multiple virulence factors that enable the bacterium to evade host immune defenses, invade tissues, and disseminate systemically.These mechanisms are particularly pronounced in hypervirulent strains and constitute the basis for invasive diseases such as pyogenic liver abscess.

CPS is the most critical virulence factor of *K. pneumoniae*. The capsule confers strong serum resistance by inhibiting complement activation, preventing C3b deposition, and blocking formation of the membrane attack complex (20). The transcriptional regulators RmpA and RmpA2 positively regulate capsular polysaccharide synthesis, leading to capsule overproduction (21). Rather than directly modulating immune signaling, the capsule primarily functions as a physical barrier that impedes immune recognition and limits contact between immune cells and bacterial surface structures, thereby reducing phagocytic clearance (22). Host metabolic signals play an important role in regulating virulence phenotypes in hypervirulent *K. pneumoniae*. Lactate can relieve transcriptional repression of CPS biosynthesis mediated by the cyclic adenosine monophosphate (cAMP) receptor protein by inhibiting the mannose-specific phosphotransferase system and reducing intracellular cAMP levels (23). Arginine primarily regulates the mucoid phenotype of *K. pneumoniae* through its receptor protein ArgR, which binds to the promoter region of the rmp operon to modulate rmpD expression (24). Together, these regulatory pathways highlight the high adaptability of hvKP to the host metabolic microenvironment.

Iron plays a central role in the growth and pathogenicity of *K. pneumoniae*, making it a primary target of host nutritional immunity aimed at restricting microbial access to essential nutrients (25). To overcome host-imposed iron restriction mediated by molecules such as hepcidin, transferrin, and lactoferrin, *K. pneumoniae* has evolved multiple high-efficiency iron acquisition systems, among which siderophore-mediated uptake is particularly critical (26). *K. pneumoniae* produces four major classes of siderophores—enterobactin, salmochelin, yersiniabactin, and aerobactin—which chelate ferric iron with exceptionally high affinity and are internalized via TonB-dependent transporters, thereby bypassing host iron sequestration mechanisms (27). To counteract the host innate immune protein lipocalin-2, which neutralizes enterobactin, *K. pneumoniae* glycosylates enterobactin to generate salmochelin or employs structurally distinct yersiniabactin, thereby achieving immune evasion (28, 29). Aerobactin production is strongly associated with the hypervirulent phenotype of *K. pneumoniae* (30). Meanwhile, large virulence plasmids frequently harbor gene clusters encoding aerobactin, salmochelin, and the capsule regulators regulator of mucoid phenotype A and A2 (rmpA/rmpA2), enabling coordinated enhancement of siderophore production and capsule synthesis (31). These virulence plasmids can also recombine with or coexist alongside antimicrobial resistance plasmids, facilitating the emergence and dissemination of hybrid hypervirulent and carbapenem-resistant *K. pneumoniae* strains (32). Overall, siderophores represent not only the primary mechanism by which *K. pneumoniae* overcomes host nutritional immunity but also a critical determinant of immune evasion, tissue colonization, and systemic infection.

Lipopolysaccharide (LPS) is a major component of the outer membrane of *K. pneumoniae* and consists of lipid A, a core oligosaccharide, and the O antigen, playing a crucial role in immune evasion. While canonical LPS elicits strong host inflammatory responses, some *K. pneumoniae* strains reduce immune recognition by modifying lipid A or masking LPS epitopes with the capsule, thereby attenuating inflammation and decreasing bacterial clearance (33). Distinct LPS O-antigen serotypes exhibit functional differences in complement resistance and inflammatory modulation; notably, O1-expressing strains interfere with the proper insertion and polymerization of complement membrane attack complex component C9 on the bacterial membrane, thereby evading complement-mediated killing (34). In contrast, multidrug-resistant *K. pneumoniae* strains expressing the O2afg (and, to a lesser extent, O2a) antigen markedly suppress the production of proinflammatory cytokines and chemokines by monocytes and inhibit nuclear translocation of nuclear factor kappa B (NF-κB), thereby evading early inflammatory immune responses and promoting systemic dissemination (35). Collectively, structural diversity of the lipopolysaccharide O antigen shapes the immune evasion capacity and pathogenic potential of *K. pneumoniae* by modulating complement activation and host inflammatory signaling.

Outer membrane vesicles (OMVs) are nanoscale vesicles actively released by *K. pneumoniae* under both growth and stress conditions and are enriched in LPS, outer membrane proteins, periplasmic and cytoplasmic proteins, virulence and resistance factors, nucleic acids, and metabolites, serving as important vehicles for virulence and immune evasion (36, 37). *K. pneumoniae* delivers effector molecules to epithelial cells and macrophages via OMVs, thereby activating or reshaping inflammatory responses, inducing apoptosis or pyroptosis (38, 39), and mediating horizontal gene transfer of virulence and resistance plasmids, which promotes the emergence and dissemination of hypervirulent carbapenem-resistant *K. pneumoniae* (40).

Outer membrane protein A (OmpA) is a representative virulence-associated protein of *K. pneumoniae* that enhances bacterial survival in the host by promoting adhesion, increasing serum resistance, and stabilizing outer membrane integrity. In addition, OmpA is frequently delivered to host cells via OMVs, where it interferes with inflammatory signaling and induces cellular damage, thereby impairing innate immune clearance and facilitating immune evasion (41, 42).

HvKP has a more complex pathogenic mechanism than classical *K. pneumoniae*, with stronger invasiveness and immune evasion capabilities. Although hypervirulent *K. pneumoniae* strains are generally more susceptible to antibiotics than classical strains, their enhanced invasiveness and immune evasion largely depend on excessive capsule production, a broader siderophore repertoire, and reduced induction of host inflammatory responses (43).

**Fig 1:**
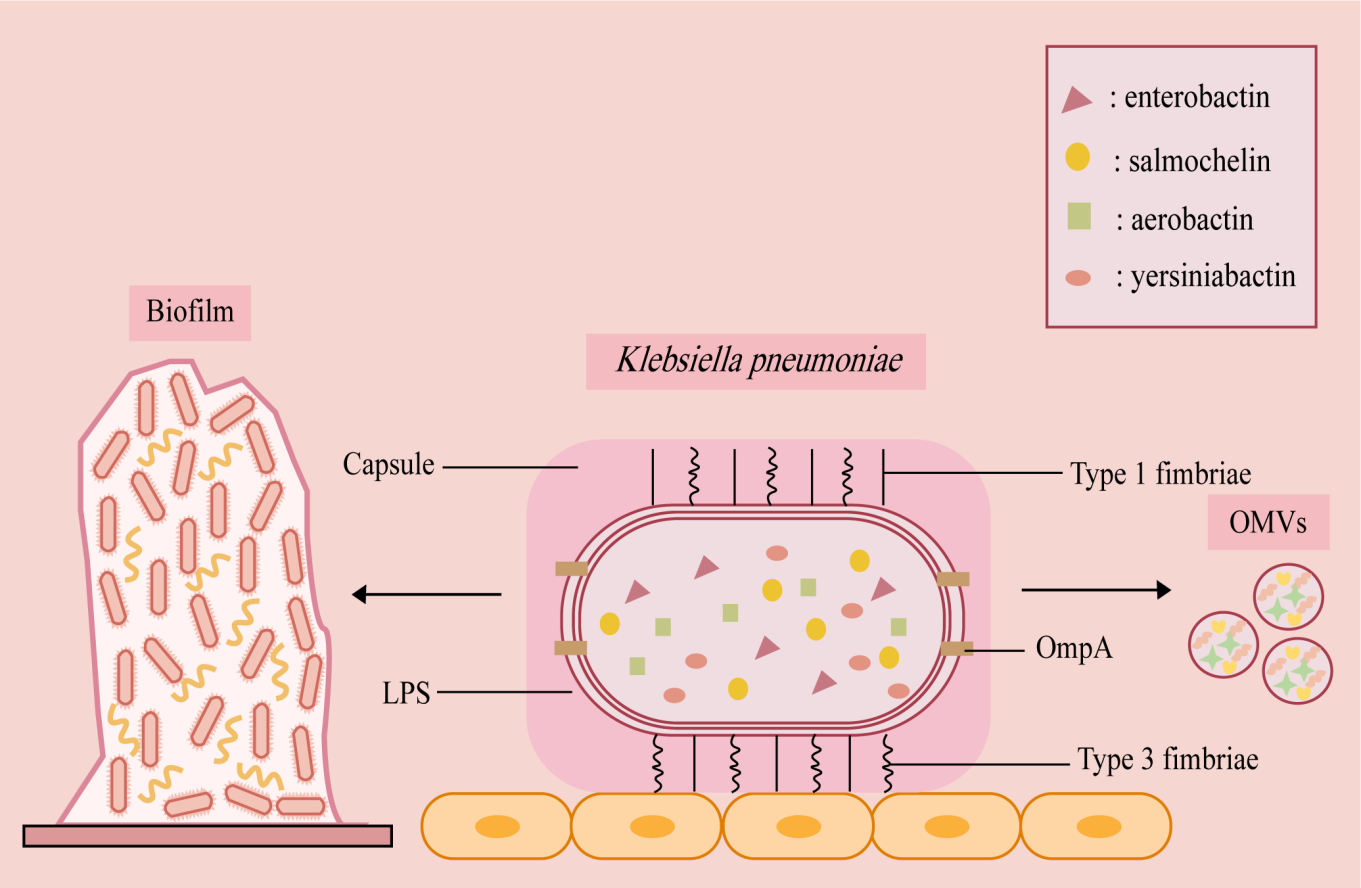
*K. pneumoniae* achieves stable adhesion and colonization, enhanced pathogenicity, and immune evasion through the coordinated action of multiple virulence factors. Type I and type III fimbriae mediate adhesion to host epithelial cells and biofilm formation, respectively, thereby promoting persistent bacterial survival within the host. Although capsular polysaccharide (CPS) is not a primary adhesin, it enhances colonization stability by increasing serum resistance and inhibiting phagocytosis. In addition, siderophore-mediated iron acquisition enables the bacterium to overcome host nutritional immunity, while structural modification of the lipopolysaccharide (LPS) O antigen modulates complement activation and host inflammatory signaling pathways. Outer membrane protein A further strengthens bacterial adhesion, serum resistance, and immune evasion. Moreover, outer membrane vesicles (OMVs) deliver multiple virulence factors to host cells, reshape inflammatory responses, and facilitate the horizontal transfer of virulence and antibiotic resistance genes.

Together, these virulence traits act synergistically to promote persistent intestinal colonization, evade immune clearance, and facilitate dissemination along the gut-liver axis, thereby exacerbating disease severity (Fig. 1).

## BASIC MECHANISM OF THE GUT-LIVER AXIS

### Physiological functions of the gut-liver axis

The gut-liver axis refers to the bidirectional regulatory mechanism formed between the intestine and the liver through portal vein, bile system, and neuroendocrine signal connections. It plays a central role in maintaining body metabolism, immune homeostasis, and toxin clearance. The anatomical connection between the two is mainly reflected in three aspects: blood circulation, neural connection, and immune regulation.

First, from the perspective of blood circulation, the intestine directly transports digested and absorbed nutrients, microbial metabolites, toxins, and microbe-associated molecular patterns (MAMPs) to the liver through the portal venous system. The liver regulates the metabolic balance of the whole body by further metabolizing and detoxifying these substances (44). At the same time, the liver maintains dynamic balance with the intestine through the bile system. Bile not only participates in lipid metabolism but also plays an important antibacterial role, inhibiting the over-proliferation of harmful bacteria in the intestine. Intestinal flora metabolizes part of primary bile acids into secondary bile acids. These molecules reverse regulate metabolism and inflammatory responses in the liver and intestine through signaling pathways such as farnesoid X receptor (FXR) and bile acid receptor TGR5 (45).

Second, neural connections achieve two-way communication through the vagus nerve and the intestinal nervous system. The status information of the intestine can regulate the liver’s metabolic activities and immune responses through neural signals, such as the regulation of lipid and sugar metabolism. At the same time, the liver’s neuroregulatory effect also helps promote the stability of the intestinal barrier (46).

Finally, from the perspective of the immune system, the gut and liver form a network that integrates immune perception and response. The intestinal mucosa structure is the first barrier that protects the steady state of the intestinal liver axis. It is composed of intestinal epithelial cells and tight junction proteins (such as Claudin and Occludin), preventing bacteria and toxins from entering the blood through the intestinal wall (47). At the same time, intestinal flora metabolizes short-chain fatty acids (SCFAs), vitamins, and other beneficial factors, maintains the integrity of the intestinal epithelial barrier, and regulates local and systemic immune responses by interacting with the intestinal immune system (48). In addition, intestinal mucosal defense is further enhanced by secreted immunoglobulin A (sIgA) and antibacterial peptides (49). The portal vein transports small molecules of nutrients and trace amounts of bacterial proteins to the liver. This process is monitored by the inherent immune system in the liver (such as Kupffer cells and liver macrophages).

These immune cells can recognize MAMPs and quickly eliminate gut-derived harmful substances, effectively inhibiting the occurrence of systemic inflammation (50).

### Gut-liver axis and different inflammatory diseases

In various inflammatory diseases, gut-liver axis disorders contribute through specific mechanisms and pathways. Their pathological characteristics depend on the disease type, the nature of microbial imbalances, and the immune system’s response.The dysregulation of intestinal flora, metabolites, and barrier functions, along with altered liver immune responses, collectively forms a distinct pathological network underlying various diseases (Table 1).

**TABLE 1:** Inflammatory diseases

Disease	Gut microbiota and barrier alterations	Inflammatory mechanisms	Pathological characteristics	Reference
Inflammatory Bowel Disease (IBD)	Gut microbiota dysbiosis: Reduced diversity (e.g., beneficial bacteria like Lactobacilli & Bifidobacteria decrease) and increased pathogenic bacteria (e.g., *E. coli*) Impaired gut barrier: Increased intestinal permeability, promoting bacterial translocation and systemic inflammation	Immune dysregulation: Excessive activation of T cells and B cells with elevated pro-inflammatory cytokines like TNF-α, IL-1β, and IL-6. Pyroptosis further aggravates local and systemic inflammation	Crohn’s Disease (CD):Transmural inflammation, intestinal wall thickening, strictures, and fistulae. Ulcerative Colitis (UC): Mucosal and submucosal ulceration confined to the colon and rectum	(51–53)
Autoimmune liver disease (AILD)	Altered gut microbiota: Decrease in beneficial bacteria and increases in harmful bacteria. Increased gut permeability facilitates translocation of endotoxins, triggering liver immune responses	Overactivation of CD4+ and CD8+ T cells, releasing TNF-α and IFN-γ. Hepatic macrophages contribute to inflammation and fibrosis	Chronic liver inflammation, hepatocyte necrosis, fibrosis, and cirrhosis. Histological "rosette" formations and interface lymphocyte infiltration with progression to hepatocellular carcinoma in severe cases	(54–56)
Alcoholic Liver Disease (ALD)	Alcohol disrupts gut microbiota: Reduced beneficial bacteria and overgrowth of harmful bacteria. Gut barrier damage: Alcohol metabolism generates toxic intermediates (e.g., acetaldehyde) damaging epithelial cells	Alcohol-induced oxidative stress activatesinflammatory pathways: TLR4-mediated immune activation by endotoxins. NLRP3 inflammasome activation worsens inflammation and hepatocyte injury	Fatty liver, inflammation, fibrosis, and hepatocytenecrosis. Histopathology reveals lobular steatosis, neutrophilic infiltrates, and perivenular fibrosis	(57–60)
Non-alcoholic Fatty Liver Disease (NAFLD)	Gut dysbiosis linked to metabolic syndrome: Reduced SCFAs and overproduction of endotoxins. Compromised gut barrier increases translocation of harmful substances into portal circulation	Chronic low-grade inflammation driven by immune cell infiltration (e.g., macrophages). Elevated TNF-α and IL-6 levels promote progression to non-alcoholic steatohepatitis (NASH)	Hallmark is hepatic steatosis in NASH, inflammation, hepatocyte ballooning, and fibrosis occur. Advanced disease may lead to cirrhosis or hepatocellular carcinoma	(61–63)
Primary sclerosing cholangitis (PSC)	Reduced Firmicutes (e.g., butyrate-producing bacteria) and increased Proteobacteria Downregulation of tight junction proteins (e.g., occludin), leading to bacterial translocation Bile acid dysmetabolism: Decreased secondary bile acids (e.g., deoxycholic acid) and suppressed FXR signaling	Innate immune activation: TLR4 recognizes LPS, triggering Kupffer cells to release TNF-α and IL-6 Adaptive immune dysregulation: Th17 cell infiltration and IL-17-driven fibrotic pathways Bile acid toxicity: Accumulation of hydrophobic bile acids (e.g., CDCA) activating hepatic stellate cells	Bile duct fibrosis: "Onion-skin" concentric periductal fibrosis with strictures/obliteration Hepatic injury: Progressive loss of small bile ducts, portal inflammation, cholestasis, and cirrhosis Gut-liver axis link: 60%–80% comorbidity with IBD, primarily ulcerative colitis	(64–66)

Growing evidence indicates that Inflammatory Bowel Disease (IBD), Non-alcoholic Fatty Liver Disease(NAFLD), and Primary sclerosing cholangitis (PSC) are closely associated with *K. pneumoniae* infection. Studies have shown that *K. pneumoniae* is significantly enriched in the fecal microbiota of patients with IBD and that its abundance correlates with disease exacerbation. Further analyses revealed that the expansion of IBD-associated antibiotic resistance genes (ARGs) and mobile genetic elements (MGEs) is largely driven by the increased abundance of *K. pneumoniae* and other members of the Enterobacteriaceae family, suggesting that these IBD-associated pathobionts may more readily acquire and maintain antibiotic resistance (67). Studies have shown that high alcohol-producing strains of *K. pneumoniae* can generate substantial amounts of endogenous ethanol in the gut via the 2,3-butanediol fermentation pathway, which is subsequently delivered to the liver through the portal circulation, thereby inducing hepatic steatosis, oxidative stress, and inflammation and ultimately leading to NAFLD in the absence of exogenous alcohol consumption (68, 69). *K. pneumoniae* is significantly enriched in patients with PSC and exhibits pronounced pathogenic potential. *K. pneumoniae* isolates derived from the intestinal tract of PSC patients harbor multiple virulence-associated genes related to adhesion, iron acquisition, and the type VI secretion system, display enhanced epithelial adherence, and are capable of inducing inflammatory responses in biliary or intestinal epithelial cells (70, 71). Although direct causal evidence linking *K. pneumoniae* to other chronic inflammatory or metabolic disorders remains limited, the altered gut-liver microenvironment shaped by persistent inflammation, microbial dysbiosis, and impaired immune function may facilitate its colonization, expansion, and translocation along the gut-liver axis. Collectively, these findings suggest that *K. pneumoniae* acts not as a disease-specific pathogen but rather as a key pathobiont in the context of gut-liver axis dysregulation, with its pathogenic impact being highly dependent on host inflammatory status, metabolic conditions, and immune responses.

## EFFECT OF *K. PNEUMONIAE* ON INTESTINAL BARRIER

### Composition and function of the intestinal barrier

The intestinal barrier plays a crucial role in maintaining gut health and overall bodily balance. Its primary function is to block harmful substances and pathogens while effectively absorbing nutrients. The intestinal barrier is made up of several components, including intestinal epithelial cells, tight junctions, the mucus layer, and microbiota. Intestinal epithelial cells consist primarily of absorptive, goblet, and Paneth cells. Absorbing cells are the main component of the intestinal epithelium and are responsible for absorbing nutrients and water. Goblet cells secrete mucus, lubricating the gut and protecting epithelial cells from stimulation. Paneth cells secrete antibacterial substances and digestive enzymes and participate in immune defense (72). Tight junctions form a physical barrier between cells, regulating material permeability and preventing the invasion of harmful substances and pathogens (73, 74). Damage to the intestinal barrier can increase permeability, leading to "leaky gut" and health issues like enteritis and irritable bowel syndrome (75, 76).

The mucus layer plays a crucial role in maintaining the intestinal barrier. Its primary component consists of mucins. Additionally, it contains antimicrobial peptides, immunoglobulins (such as sIgA), and various carbohydrates. Together, these components create the biological barrier of the intestine, effectively safeguarding intestinal epithelial cells from pathogens and harmful substances (77). Studies have shown that the chemical structure and composition of mucus can be influenced by multiple factors, including diet, composition of the microbial community, and the immune status of the host (78). For example, healthy gut microorganisms can promote the production of mucus and maintain its integrity, thereby enhancing the function of the gut barrier (79). A complex interaction exists between the mucus layer and intestinal microorganisms, wherein mucus provides a habitat for these microorganisms while also regulating community composition and function (79). Studies have shown that a healthy microbial community can promote mucus production and secretion and enhance the integrity of the mucus layer (80). At the same time, specific microorganisms (such as Akkerman siamuciniphila) can break down mucins and release metabolic products such as short-chain fatty acids. These substances not only help maintain intestinal barrier function but also regulate the host’s immune response (81).

Gut-associated lymphoid tissue (GALT) is an important part of the intestinal immune system, mainly including structures such as Peyer’s spots, isolated lymph follicles, and intestinal lymph nodes in the intestine. Research indicates that GALT produces specific antibodies, such as IgA, and also influences the intestinal immune response by regulating the intestinal microbiota’s composition (82). Additionally, immune cells in GALT, including dendritic cells, T cells, and B cells, interact with gut epithelial cells to enhance the integrity and function of the intestinal barrier (83). The interaction between immune cells and the gut barrier plays a vital role in maintaining intestinal health. Gut epithelial cells regulate the recruitment and activation of immune cells by producing cytokines and chemokines, thereby forming a dynamic immune environment (84). For instance, dendritic cells in the gut recognize intestinal microorganisms and food antigens. They activate specific immune responses by presenting these antigens to T cells (85). At the same time, immune cells such as macrophages and natural killer cells also directly affect the function and integrity of intestinal epithelial cells by secreting cytokines and chemokines. This interaction not only helps resist invasion by pathogens but also promotes the repair and regeneration of the intestinal barrier (86).

### Translocation and invasion of *K. pneumoniae*

The mucus layer is the first barrier of intestinal defense. Infection with *K. pneumoniae* can cause significant changes in the structure and function of the mucus layer. After bacterial infection, the thickness and integrity of the mucus layer may be compromised, resulting in a decrease in intestinal barrier function, thereby increasing the risk of pathogen invasion (87). Additionally, *K. pneumoniae* may promote the growth of harmful bacteria in the intestine, which further damages the mucus layer’s integrity and increases intestinal permeability (19).

There are studies that infected zebrafish with different *K. pneumoniae* strains to explore the effect of bacteria on their intestines. All three strains caused intestinal damage, leading to changes in microbial diversity. This resulted in a decrease in beneficial bacteria and an increase in harmful bacteria. Among these, the hypervirulent, drug-resistant strain significantly reduces the number of goblet cells, damages the epithelial mucosa, and increases bacterial colonization (88). *K. pneumoniae* binds to integrins on intestinal epithelial cells, enhancing its ability to attach and damaging the cell skeleton, which facilitates bacterial invasion (89). *K. pneumoniae* secretes various toxins and enzymes that interfere with host cell signal transduction pathways, further enhancing its invasion ability. For example, certain strains of *K. pneumoniae* can upregulate the activity of the transforming growth factor beta (TGF-β) signaling pathway, thereby promoting cell adhesion and invasion (90). Studies have found that *K. pneumoniae*, through its virulence factors, shows greater invasiveness and translocation ability when intestinal barrier function is impaired (such as loss of MyD88 signal). The mechanism of its destruction of intestinal barrier includes decreased antibacterial activity, imbalance of flora, and increased permeability of mucosal barrier (91).

Once the intestinal barrier is compromised, the hypervirulent *K. pneumoniae* that inhabit the gastrointestinal tract can translocate, breach the intestinal barrier, and reach the liver via the hepatic portal vein. *K. pneumoniae* enhances the activity of macrophage-derived gelatinase in the colon, leading to intestinal barrier dysfunction. It also significantly drives inflammation, fibrosis, and carcinogenic signaling by colonizing the liver after intestinal metastasis (92). During this process, the sap protein associated with iron transport in *K. pneumoniae* promotes bacterial translocation across the Caco2 intestinal epithelial monolayer, macrophage adhesion and phagocytosis, and induction and release of pro-inflammatory cytokines, and increases liver bacterial load and liver abscess shape in a mouse gastrointestinal infection model (93). The Type VI secretion system in *K. pneumoniae* aids in the adhesion and invasion of Caco-2 cells, while also regulating the strain’s virulence, facilitating intestinal colonization, and promoting bacterial dissemination (94, 95). Disruption of the intestinal microbiota (due to antibiotic administration) diminishes the expression of IL-22, consequently reducing the levels of the antimicrobial peptide Reg3β, thereby impairing the ability of hvKP to migrate from the gut to the liver (96).

**Fig 2:**
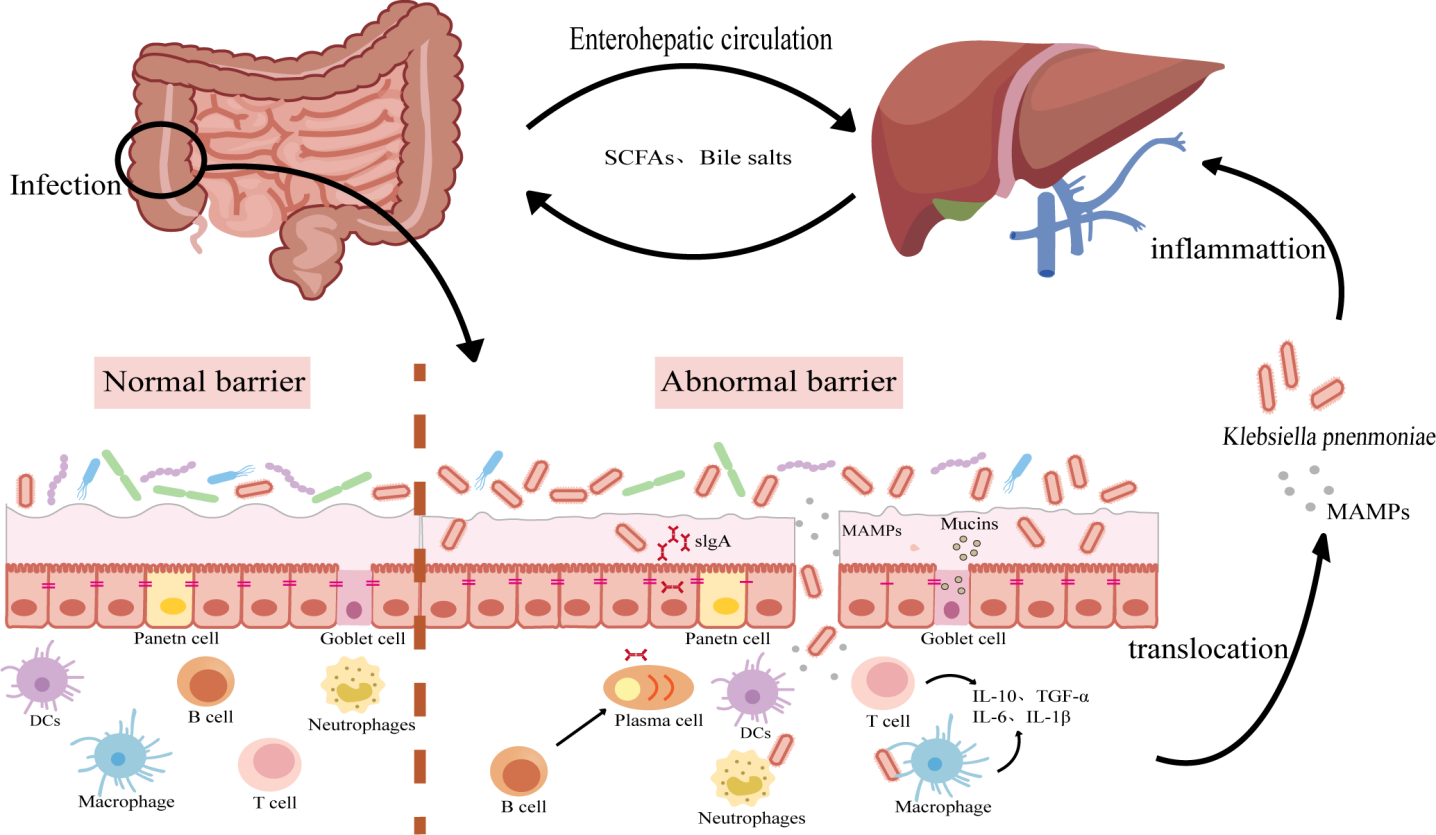
Disruption of the intestinal barrier and bacterial translocation. *K. pneumoniae* infection (especially hvKp) damages the intestinal mucus layer, reduces goblet cells, and alters microbial communities, thereby increasing intestinal permeability. The bacteria adhere to integrins on epithelial cells, disrupt the cytoskeleton, and exploit virulence factor. Impairment of epithelial defenses (e.g., reduced antimicrobial activity, altered flora, and loss of tight junction proteins including ZO-1 and occludin) further enhances translocation. Once the barrier is breached, *K. pneumoniae* crosses into the portal circulation and colonizes the liver, where it activates macrophages and neutrophils, drives inflammatory and fibrotic signaling, and promotes abscess formation.

*K. pneumoniae* typically triggers an inflammatory response in the liver following translocation, potentially resulting in localized suppuration. In severe cases, it can disturb the liver’s immune balance, leading to the spread of infection to other organs or the bloodstream (97). Therefore, effectively eliminating *K. pneumoniae* and its virulence factors has become crucial during the intestinal-liver axis metastasis. The mobilization of immune cells and activation of inflammatory pathways in both the intestine and liver are critical to this process (Fig. 2).

## IMMUNE REGULATION OF INTESTINAL LIVER AXIS UNDER *K. PNEUMONIAE* INFECTION

### Immune cells and inflammatory mediators

*K. pneumoniae* pneumonia causes significant changes in the quantity and function of various immune cells in the intestinal tract, including T cells, B cells, macrophages, dendritic cells, and natural killer cells. If bacterial translocation or hematogenous dissemination occurs, hepatic cells respond quickly to maintain overall immune balance. However, *K. pneumoniae* infection can cause hepatic immune dysfunction, leading to liver injury and systemic inflammation, which may worsen the prognosis. Macrophages are the main effector cells in the immune response against *K. pneumoniae* infection and are usually activated by the IL-36 pathway to prevent the bacteria from colonizing (98). Macrophages have the ability to ingest and digest pathogens to eliminate infections directly, as well as to enhance the host’s defense mechanisms by producing antimicrobial peptides and reactive oxygen species (99). Additionally, macrophages can modulate inflammatory responses by secreting various cytokines and chemokines, such as IL-6, IL-1β, TNF-α, CCL2, and CXCL2, to recruit and activate more immune cells (100, 101). Research indicates that the polarization state of macrophages (such as M1 and M2) significantly influences the outcome of infections, with M1 macrophages effectively clearing pathogens, while M2 macrophages may promote immune tolerance and repair processes (102, 103). The depletion of macrophages can result in compromised intestinal barrier function, evidenced by decreased expression of tight junction proteins like ZO-1 and Occludin, thereby enhancing intestinal permeability and promoting bacterial translocation (101).

In the initial phase of infection, neutrophils swiftly accumulate at the infected site. They release pro-inflammatory cytokines to boost the local inflammatory response. Additionally, they produce myeloperoxidase (MPO) and reactive oxygen species (ROS) to eliminate pathogens (104). The buildup of neutrophils in the liver worsens local inflammation and tissue damage, leading to the formation of liver abscesses (105). Hepatic Kupffer cells can recognize *K. pneumoniae* that has translocated from the intestines using specific pattern recognition receptors (PRRs). This recognition triggers the release of inflammatory cytokines. These inflammatory signals then return to intestinal immune tissues, such as Peyer’s patches, further stimulating local immune responses (106, 107).

*K. pneumoniae* infection activates TH17 cells in the liver and mesenteric lymph nodes, leading to the release of IL-17. This contributes to liver inflammation and damage to the bile ducts (108). Additionally, antigens are presented through bile or the bloodstream (109). The gut-liver axis influences naive B cells, activating them in the intestines to produce IgA, which strengthens the integrity of the intestinal barrier (110). Certain immune cell types in the gut and liver, including natural killer (NK) cells and NKT cells, may also be activated to help evade the host’s immune response to bacteria (111).

### Pathway of inflammation

Intestinal inflammation affects liver metabolism and immune function by releasing inflammatory mediators, which worsen liver inflammation. Inflammatory signaling pathways play a crucial role in regulating immune responses and inflammation. During *K. pneumoniae* infection of intestinal epithelial cells, increased TLL1 expression activates the TGF-β signaling pathway, which enhances bacterial adhesion and invasion. A transcriptomic analysis of *K. pneumoniae* infection in Caco2 cells revealed significant enrichment of the TNF signaling pathway. Additionally, it showed upregulation of calcium-binding genes, such as TNNC2 and CAPN3, in the calcium ion signaling pathway, indicating their role in inflammatory responses (90). Through the activation of the MyD88-NF-κB pathway by TLR4 in intestinal epithelial cells, *K. pneumoniae* promotes the secretion of cytokines including IL-6 (108, 112). The *K. pneumoniae* strains isolated from bloodstream infections activate the Rho GTPase and PI3K/AKT signaling pathways in intestinal epithelial cells, inducing cytoskeletal rearrangement, thereby facilitating bacterial phagocytosis and invasion (113).

In the context of existing literature, it is observed that the inflammatory response triggered by *K. pneumoniae* primarily originates from macrophages. *K. pneumoniae* infection induces SARM1 expression in macrophages. The bacteria’s virulence factors, such as LPS and capsule, activate the TLR4 pathway in cells, leading to type I interferon generation through the TRAM-TRIF-IRF3 axis. This type I interferon upregulates SARM1 expression, activating the p38-MAPK signaling pathway to release IL-10, which exhibits anti-inflammatory properties. Furthermore, SARM1 inhibits the AIM2 inflammasome activation induced by *K. pneumoniae* and the subsequent IL-1β secretion (114, 115). The infection by *K. pneumoniae* is capable of regulating the Gas6-Axl signaling pathway within macrophages, resulting in the augmentation of intestinal ZO-1 and occludin expression, which, in turn, hinders bacterial penetration of the intestinal epithelium and safeguards against its migration to the liver (116). Hypervirulent *K. pneumoniae* inhibits the cathepsin B-NLRP3-Caspase1 pathway, reducing macrophage pyroptosis. This may relate to immune evasion strategies provided by its capsule or other virulence factors (117). In cases of hypervirulent *K. pneumoniae* infection, macrophages activate the SYK-IRG1-itaconate pathway to stimulate IRG1 expression, facilitating itaconate production. Activation of the SYK signaling pathway enhances cytokine secretion; subsequently, itaconate exhibits anti-inflammatory effects by inhibiting the SYK pathway through negative feedback (100). *K. pneumoniae* also activates the PI3K/AKT/Rab14 axis in macrophages, inhibiting phagosome-lysosome fusion and preventing phagosome maturation, thereby ensuring intracellular survival. This mechanism has been confirmed only *in vitro*, and no *in vivo* experiments have been conducted (118). However, this finding provides new insights into the immune evasion and bacterial translocation of *K. pneumoniae* within the host gut-liver axis.

**Fig 3:**
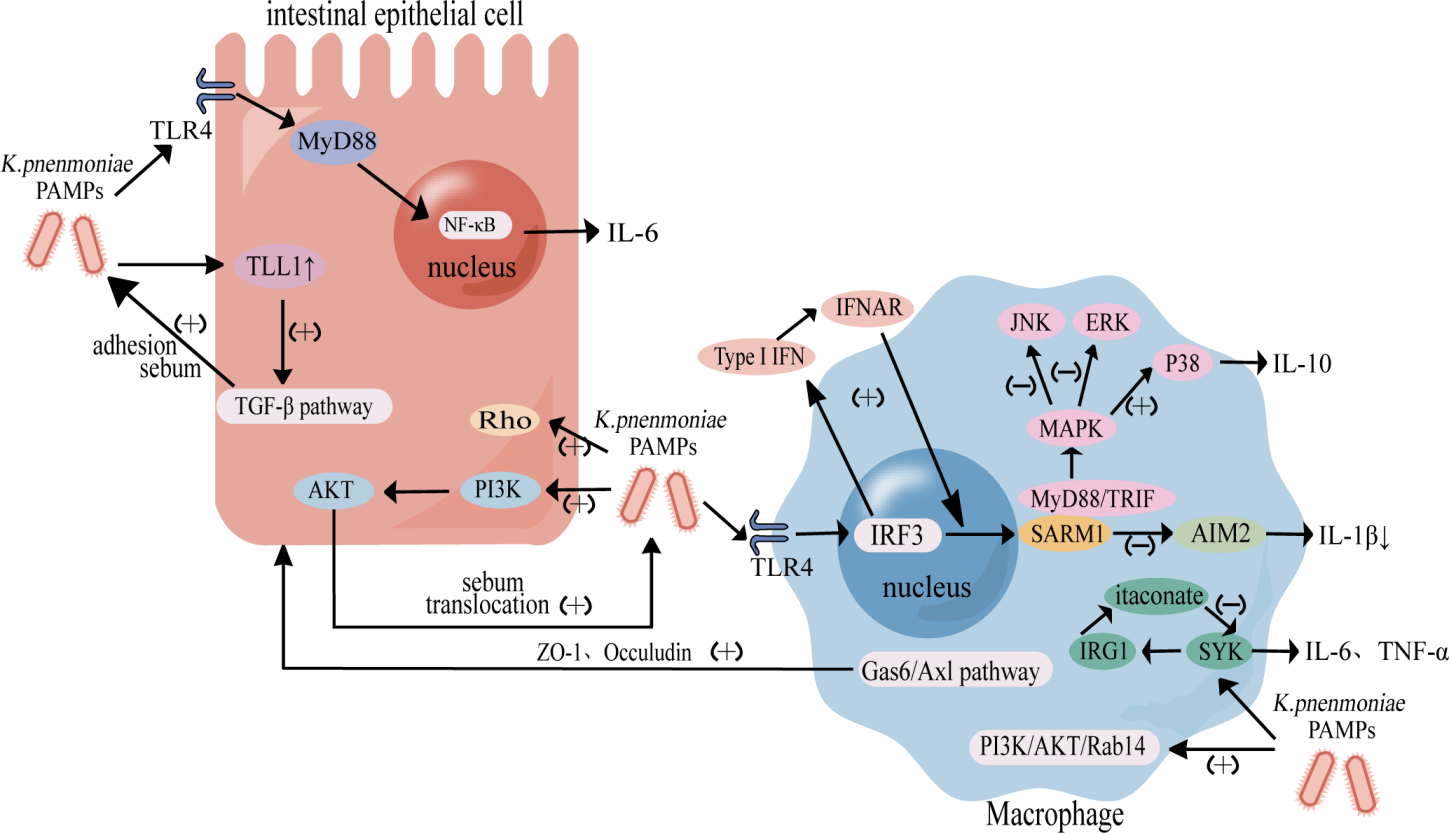
*K. pneumoniae* orchestrates intestinal inflammation through the coordinated activation and modulation of multiple signaling pathways in intestinal epithelial cells and macrophages. In intestinal epithelial cells, infection induces the expression of TLL1 and activates the TGF-β signaling pathway, enhancing bacterial adhesion and invasion. Activation of the TLR4-MyD88-NF-κB signaling pathway promotes the secretion of pro-inflammatory cytokines, including IL-6, while the Rho GTPase and PI3K/AKT signaling pathways drive cytoskeletal rearrangement, facilitating bacterial adhesion and invasion. In macrophages, *K. pneumoniae* activates TLR4-dependent type I interferon signaling through the TRAM-TRIF-IRF3 axis, upregulating SARM1 expression and triggering the p38-MAPK pathway, which promotes IL-10 release and suppresses AIM2 inflammasome-mediated IL-1β secretion. Additional regulatory mechanisms include the modulation of the Gas6-Axl signaling pathway to enhance intestinal tight junction integrity, activation of the SYK-IRG1-itaconate pathway, and negative feedback regulation of inflammation. Moreover, the PI3K/AKT/Rab14 axis is activated to inhibit phagosome-lysosome fusion, ensuring bacterial survival within the cell. Collectively, these signaling pathways contribute to immune dysregulation, barrier dysfunction, and promote immune evasion and bacterial translocation along the gut-liver axis.

Collectively, these findings indicate that *K. pneumoniae* orchestrates intestinal inflammation through coordinated activation and modulation of multiple signaling pathways in both epithelial cells and macrophages, thereby reshaping immune responses, impairing barrier integrity, and facilitating immune evasion and bacterial translocation along the gut-liver axis (Fig. 3).

## FUTURE DIRECTIONS

Despite substantial progress in understanding the virulence traits of *K. pneumoniae*, particularly hypervirulent strains, fundamental questions regarding host-pathogen interactions along the gut-liver axis remain unresolved. Addressing these issues will be essential for developing a more integrated and translational framework of hvKP pathogenesis.

First, the precise mechanisms by which the key virulence determinants of Klebsiella pneumoniae—including excessive CPS production, siderophore-mediated iron acquisition, LPS, and OMVs-associated effector molecules—cooperate to regulate the immune network along the gut-liver axis remain poorly understood. Most current studies focus on individual virulence factors or isolated host pathways, and the mechanistic link between the two has not been clearly established, often due to the reliance on reductionist *in vitro* systems. Future studies should adopt integrated *in vivo* approaches that combine animal models of intestinal colonization with liver dissemination, enabling simultaneous assessment of epithelial barrier integrity, immune cell activation, and bacterial translocation. Emerging platforms such as intestinal and hepatic organoids, as well as gut-liver-on-a-chip systems, may further facilitate the dissection of spatiotemporal host-pathogen interactions under physiologically relevant conditions (119).

Second, the dynamic immune regulatory mechanisms of the gut-liver axis during *K. pneumoniae* infection are insufficiently characterized. Most available data primarily reflect static immune responses during infection, limiting insight into the temporal evolution of immune activation, tolerance, and exhaustion. Future investigations employing time-resolved immune profiling, single-cell transcriptomics, and spatial transcriptomic technologies may help elucidate how innate and adaptive immune populations are sequentially engaged across the intestinal mucosa and the liver.

Third, the interplay between host immune heterogeneity and bacterial virulence diversity in shaping clinical outcomes remains largely unexplored. Host factors such as metabolic status, underlying liver disease, and microbiota composition are likely to influence susceptibility to *K. pneumoniae* invasion and dissemination. These host-pathogen interactions are likely to determine whether infection remains localized in the intestine, progresses to liver abscess, or disseminates systemically, influencing disease severity and prognosis. Integrating clinical cohort studies with experimental infection models and multi-omics analyses may enable stratification of host risk profiles and identification of immune signatures associated with severe disease. This integrative strategy could also inform the development of host-directed therapeutic interventions targeting immune pathways rather than bacterial viability alone.

Collectively, addressing these outstanding questions will require interdisciplinary efforts that bridge microbiology, immunology, and systems biology. A deeper understanding of *K. pneumoniae*-mediated immune dysregulation along the gut-liver axis may ultimately facilitate the identification of novel diagnostic markers and immunomodulatory strategies for preventing invasive *K. pneumoniae* infections.

## CONCLUSION

This review systematically summarizes the mechanisms of the gut-liver axis during *K. pneumoniae* infection, with a focus on discussing the typical virulence factors of *K. pneumoniae*, and the key processes through which *K. pneumoniae* disrupts the intestinal barrier, activates inflammatory signaling pathways, and regulates immune responses. Research indicates that the virulence factors of *K. pneumoniae*, along with disturbances in the gut microbiota and the gut-liver axis, significantly contribute to the onset and progression of infection. These findings not only deepen the understanding of the mechanisms behind *K. pneumoniae* infection but also provide a theoretical basis for developing precise therapeutic strategies.
